# Clinicopathological Characteristics and Disease Chronicity in Glomerular Diseases: A Decade-Long Study at Romania’s Largest Kidney Biopsy Reference Center

**DOI:** 10.3390/biomedicines12061143

**Published:** 2024-05-22

**Authors:** Nicolae Pană, Gabriel Ștefan, Simona Stancu, Adrian Zugravu, Otilia Ciurea, Nicoleta Petre, Gabriel Mircescu, Cristina Căpușă

**Affiliations:** 1 Department of Nephrology, “Carol Davila” University of Medicine and Pharmacy, 050474 Bucharest, Romania; pananicolaemd@gmail.com (N.P.); simonastancu2003@yahoo.com (S.S.); adzugravu@yahoo.com (A.Z.); otilia3091@yahoo.com (O.C.); nicoleta.petre@gmail.com (N.P.); gmircescu@hotmail.com (G.M.); ccalexandr@yahoo.com (C.C.); 2 “Diaverum Morarilor” Nephrology and Dialysis Medical Center, 022452 Bucharest, Romania; 3 “Dr. Carol Davila” Teaching Hospital of Nephrology, 010731 Bucharest, Romania

**Keywords:** glomerular diseases, kidney biopsy, renal histopathological prognostic score, immunoglobulin A nephropathy

## Abstract

Glomerular diseases (GDs), significant causes of end-stage kidney disease, are better understood through epidemiological studies based on kidney biopsies (KBs), which provide important insights into their prevalence and characteristics. This study aims to analyze the clinicopathological features of GDs diagnosed from 2008 to 2017 at Romania’s largest reference center. In this decade-long study, 1254 adult patients diagnosed with GDs were included. The local previously validated renal histopathological prognostic score was calculated for each KB using four histopathologic lesions: global glomerulosclerosis, tubular atrophy, interstitial fibrosis and fibrocellular/fibrous crescents. The mean patient age was 50 years, with a male predominance (57%). The primary referral reasons were nephrotic syndrome (46%), nephritic syndrome (37%), chronic kidney disease (12%), asymptomatic urinary abnormalities (4%), and acute kidney injury (1%). Immunoglobulin A nephropathy (IgAN) was the most frequently diagnosed GD (20%), aligning with frequencies reported in European registries. Diabetic glomerular nephropathy was the most common secondary GD (10%). It also presented the highest median renal histopathological prognostic score (2), indicating a poorer prognosis. Lower eGFR and higher proteinuria were independently associated with higher scores. This decade-long study highlights IgAN as the most frequent GD diagnosed by KB. Diabetic glomerular nephropathy was identified as the most common secondary GD. The renal histopathological prognostic score, notably high in diabetic glomerular nephropathy patients, was correlated with lower eGFR and higher proteinuria, underlining its clinical relevance.

## 1. Introduction

Kidney histology continues to be the gold standard for diagnosing and classifying glomerular diseases [1]. Given that glomerular diseases are a significant cause of end-stage kidney disease, epidemiological studies based on kidney biopsies provide important insights into the prevalence and characteristics of these diseases across different geographic regions. Additionally, these data facilitate the design and recruitment of clinical trials that aim to develop new preventive and therapeutic strategies [2].

In Europe, Immunoglobulin A (IgA) nephropathy appears to be the most common primary glomerular disease, followed by focal segmental glomerulosclerosis and membranous nephropathy [3]; meanwhile, lupus nephritis is the most frequently diagnosed secondary glomerular disease through kidney biopsies [3]. However, various renal biopsy reports from Europe have highlighted geographic variations in the patterns of glomerular diseases and fluctuations in their incidence rates over time. Thus, IgA nephropathy is the most commonly diagnosed glomerular disease in several European countries, including Lithuania, Northern Ireland, Croatia, Poland, the Czech Republic, Spain, and Italy [4,5,6,7,8,9,10,11,12]. Conversely, membranous nephropathy has been identified as the predominant glomerular disease in biopsy reports from a center in Turkey, while non-IgA mesangioproliferative glomerulonephritis leads in Serbia, and mesangioproliferative glomerulonephritis, with or without IgA deposition, is most frequent in reports from Germany and Romania [13,14,15,16].

In addition to diagnosis, evaluating native kidney biopsies plays an important role in predicting renal outcomes. The degree of chronic histopathological changes, which are largely irreversible, can be as important for managing glomerular nephropathies as the primary diagnosis itself [17]. Our local renal histopathological prognostic score for glomerular diseases, which assigns one point for any degree of global glomerulosclerosis, tubular atrophy, interstitial fibrosis, and fibro-cellular/fibrous crescents, was independently correlated with renal survival, beyond traditional risk factors [18].

The aim of this study is to analyze the clinicopathological characteristics of glomerular diseases diagnosed by adult kidney biopsies conducted between 2008 and 2017 at the “Dr. Carol Davila” Teaching Hospital of Nephrology, the largest kidney biopsy reference center in Romania. This center not only serves the entire southeast region of Romania, covering 14 counties and a population of 8,519,182, but also stands out as the only facility in the country to routinely utilize electron microscopy in renal pathology. Additionally, the study will elaborate on the previously validated local renal histopathological prognostic score for glomerular diseases, offering insights into the prevalence and determinants of kidney disease chronicity.

## 2. Materials and Methods

### 2.1. Study Population

We conducted a retrospective, observational study of all adults with biopsy-proven glomerular diseases from January 2008 to December 2017 in the Nephrology department of the “Dr. Carol Davila” Teaching Hospital of Nephrology, Bucharest, Romania. From a total of 1813 native kidney biopsies at our center, we excluded patients younger than 18 years old (125 patients), those diagnosed with conditions other than glomerular diseases (228 patients), those who underwent more than one biopsy during the study period (45 patients), those who underwent biopsy after the beginning of kidney replacement therapy (17 patients), and those with insufficient material for a histopathologic diagnosis (144 patients).

### 2.2. Clinical Data

Patient demographics and clinical data were retrieved form the electronic medical file through a standardized data form. The database records search was conducted by two analysts to enhance accuracy and reliability; discrepancies were resolved through consensus, ensuring a rigorous review process. Histopathological data were provided by the nephropathologist in a standardized report. After biopsy results were available, the treating nephrologist provided the final clinical diagnosis based on the European Renal Association Primary Renal Disease coding system [19].

The collected data were age, the presence of arterial hypertension (defined as a blood pressure >140/90 mmHg or the use of antihypertensive agents), inflammation (serum hemoglobin, C-reactive protein), lipid profile (serum cholesterol and triglycerides), serum albumin, eGFR calculated by the four-variable MDRD formula, proteinuria (quantified by 24 h urine collection) and hematuria. Only data at the moment of biopsy were used in analyses.

Based on these data, the following clinical syndromes were retrospectively defined: nephrotic syndrome, nephritic syndrome, chronic kidney disease (CKD), acute kidney injury (AKI) and asymptomatic urinary abnormalities/oligosymptomatic urinary abnormalities (AUA) (Table 1).

### 2.3. Pathologic Classification

**Figure 1:**
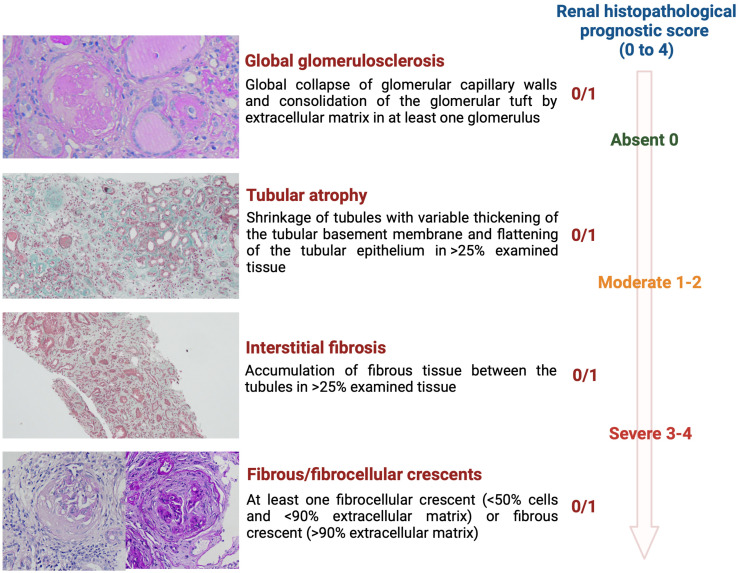
Renal histopathological prognostic score was calculated using four histopathologic lesions: global glomerulosclerosis (GS), tubular atrophy (TA), interstitial fibrosis (IF), and fibrocellular/fibrous crescents (FC). The presence of GS, TA, IF, FC was scored each with one point. The total renal histopathological chronicity score was the sum of points and ranges from 0 to 4. According to the total renal histopathological chronicity score, the overall chronic lesions were graded as “Absent” (0 total renal chronicity score), “Moderate” (1–2 total renal chronicity score), and “Severe” (3–4 total renal chronicity score). GS magnification ×400, TA magnification ×100, IF magnification ×100, FC magnification ×400.

For each biopsy specimen, light microscopy, immunofluorescence and electron microscopy were routinely performed. The local previously validated renal histopathological prognostic score was calculated using four histopathologic lesions: global glomerulosclerosis (GS), tubular atrophy (TA), interstitial fibrosis (IF), and fibrocellular/fibrous crescents (FC) [18]. The definitions of histologic variables used in our study were derived from the Mayo Clinic/Renal Pathology Society Consensus [20]. The presence of GS, TA, IF, FC was scored each with one point (Figure 1). The total renal histopathological chronicity score was the sum of points and ranges from 0 to 4 [18].

### 2.4. Statistical Analysis

Continuous variables were presented as mean with standard deviation (SD) or median with interquartile range (IQR) after testing normality with the Shapiro–Wilk test, while categorical variables were presented as percentages. Glomerular disease frequencies were shown as absolute numbers and percentages of total biopsies. Incidence rates were calculated by dividing the total number of adult native kidney biopsies diagnosed with glomerular disease from 2008 to 2017 by the total population over this period, multiplied by the duration of the study in years, to standardize the rate per million persons per year (p.m.p./year).

The association of sex, age, eGFR, proteinuria and final nephrological diagnosis with the renal histopathological prognostic score was determined in univariate and multivariable analysis by using simple and multiple linear regression models, respectively. Potential predictors were chosen based on clinical plausibility. Ninety-five-percent confidence intervals (95% CIs) were calculated for the regression coefficients, and *p*-values < 0.05 were considered significant.

Statistical analyses were performed using the SPSS program (IBM SPSS Statistics for Macintosh, Version 27.0. Armonk, NY, USA: IBM Corp) and GraphPad Prism (GraphPad Software, Version 9.5.1 (528) La Jolla, CA, USA).

### 2.5. Ethics

This study adhered to the principles of the Declaration of Helsinki, Good Clinical Practice Guidelines, and all applicable regulatory requirements. It received approval from the Ethical Committee of the “Dr. Carol Davila” Teaching Hospital of Nephrology (study reference 12/10.10.2018). All study participants or their legal representatives provided written informed consent at the time of the kidney biopsy.

## 3. Results

**Figure 2:**
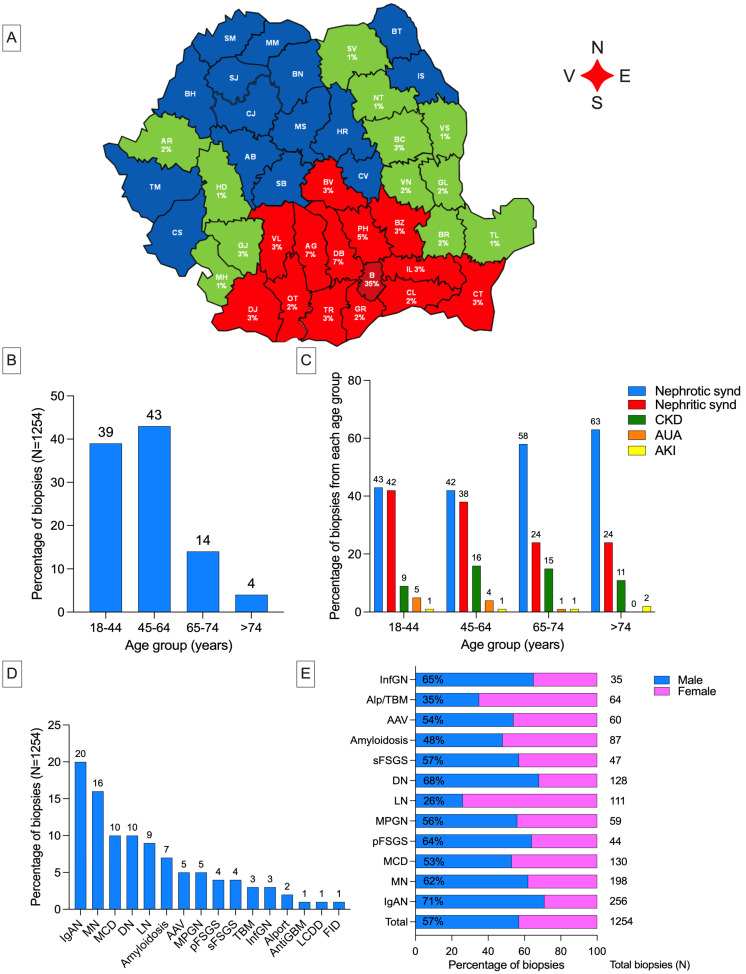
(**A**) Southeast region of Romania and the counties (in red) for which “Dr. Carol Davila” Teaching Hospital of Nephrology is a renal biopsy reference center (14 counties, 8,519,182 inhabitants); (**B**) biopsy rate according to age category; (**C**) reason for kidney biopsy per age category (percentage of kidney biopsies from each age group 18–44: 485 patients, 45–64: 539 patients, 65–74: 176 patients, >75: 54 patients); (**D**) most frequent etiology of glomerular diseases in our cohort; (**E**) sex distribution in adult patients, shown for the total number of biopsies and for individual kidney diseases. AAV, ANCA associated vasculitis; Alp, Alport syndrome; AUA, asymptomatic urinary abnormalities; AKI, acute kidney injury; CKD, chronic kidney disease; DN, diabetic glomerular nephropathy; FID, fibrillary and imunotactoid GN; FSGS, focal segmental glomerulosclerosis; GBM, glomerular basement membrane; GN, glomerulonephritis; InfGN, infection-related immune complex GN; LN, lupus nephritis; MCD, minimal change disease; MN, membranous nephropathy; MPGN, membranoproliferative glomerulonephritis.

From January 2008 to December 2017, 1254 adult native kidney biopsies diagnosed with glomerular diseases were analyzed from the southeast region of Romania (Figure 2A). The mean annual incidence rate was 14.75 cases of glomerular diseases per million persons per year. However, the overall incidence increased significantly over the years, rising from 50 cases in 2008 to 221 cases in 2017.

The mean age at biopsy was 50 years, and the biopsy rate was significantly higher among younger adults (<65 years) at 82%, compared to 18% among the elderly (Figure 2B).

The most frequent reason for kidney biopsy referral was nephrotic syndrome (46%), followed by nephritic syndrome (37%), chronic kidney disease (12%), asymptomatic urinary abnormalities (4%), and acute kidney injury (1%) (Table 2; Supplementary Table S1).

AKI was the least frequent reason for kidney biopsy, and the diagnosed glomerular diseases included diabetic kidney disease (46%), minimal change disease (28%), and pauci-immune glomerulonephritis (26%). Of the 158 patients presenting with chronic kidney disease (CKD), 25% were in stage G5, 34% in stage G4, 35% in stage G3, 4% in stage G2, and 2% in stage G1 (Supplementary Table S1).

The prevalence of nephrotic syndrome as a clinical presentation for glomerular diseases increased with age, while nephritic syndrome decreased (Figure 2C).

Males were biopsied more often (57%), although patients with a final diagnosis of lupus nephritis, Alport syndrome/thin basement membrane disease, and amyloidosis were predominantly female (74%, 65%, and 52%, respectively) (Figure 2E).

More than half of the patients had primary glomerular nephropathies (54%). The seven most frequent diagnoses, accounting for 70% of the patients studied, were, in order of frequency: IgA nephropathy (20%), membranous nephropathy (16%), minimal change disease (10%), diabetic glomerular nephropathy (10%), lupus nephritis (9%), and amyloidosis (5%) (Figure 2D).

At the time of the kidney biopsy, the median eGFR was 41.9 mL/min, proteinuria measured 3.00 g/24 h, median hematuria was 45 cells/h/HPF, and dysmorphic hematuria was observed in 48% of cases (Table 2).

In the analyzed kidney biopsies, the median number of glomeruli examined was nine. Glomerulosclerosis was the most frequent pathological lesion observed in 49% of patients, followed by interstitial fibrosis (29%), tubular atrophy (25%), and arteriolosclerosis (24%). Extracapillary proliferation was observed in at least one glomerulus in 20% of the biopsies. The median renal histopathological prognostic score was 1 (Table 2).

IgA nephropathy was the most frequently diagnosed glomerular disease via kidney biopsy, with a median diagnosis age of 44 years and a male predominance (71%). Patients most commonly presented with nephritic syndrome, while nephrotic syndrome was present in only 9% of cases. Additionally, these patients exhibited a relatively advanced stage of kidney failure, with a median eGFR of 38.4 mL/min and proteinuria of 1.5 g/24 h (Table 3).

The most common podocytopathy was membranous nephropathy (n = 198), followed by minimal change disease (n = 130) and primary focal segmental glomerulosclerosis (n = 44). Patients with membranous nephropathy and minimal change disease most frequently presented with nephrotic syndrome (92% and 82%, respectively), exhibiting a comparable severity. However, those with membranous nephropathy generally had a slightly better eGFR at diagnosis (64 vs. 56 mL/min) (Table 3).

Lupus nephritis patients were predominantly female, with a median age of 37 years, and often presented with both nephrotic and nephritic syndromes; their median eGFR was 50.2 mL/min, and they had the lowest median hemoglobin levels at diagnosis.

Diabetic glomerular nephropathy showed the most significant reduction in eGFR, at 25.9 mL/min, and was primarily characterized by nephrotic range proteinuria. It most commonly presented as chronic kidney disease (46%), followed by nephrotic syndrome (31%), nephritic syndrome (10%), and asymptomatic urinary abnormalities (9%) (Table 3).

In our cohort of glomerular disease, arterial hypertension was most commonly observed in patients with diabetic glomerular nephropathy (81%) and IgA nephropathy (70%). It was present in half of the patients with membranous nephropathy, lupus nephritis, and Alport syndrome/thin basement membrane disease. Conversely, arterial hypertension was least frequently encountered in patients with minimal change disease and amyloidosis, affecting 40% and 35% of patients, respectively (Table 3).

Diabetic glomerular nephropathy had the highest median renal histopathological prognostic score of 2, suggesting a worse prognosis, while IgA nephropathy, lupus nephritis, and amyloidosis each had a score of 1; membranous nephropathy, minimal change disease, and Alport syndrome/thin basement membrane disease had the lowest median scores of 0 (Table 3).

The association between the renal histopathological prognostic score and sex, age, eGFR, proteinuria and final nephrological diagnosis was determined using univariate and multivariable analyses (Table 4). Increasing patient age was associated with a significantly higher score in univariate analysis, but not in multivariable analysis. Lower eGFR and higher proteinuria at diagnosis were independently associated with a higher renal histopathological prognostic score. When compared with diabetic glomerular nephropathy, diagnoses of IgA nephropathy, membranous nephropathy, minimal change disease, amyloidosis, thin basement membrane/Alport syndrome and infection-related immune complex glomerulonephritis were associated with a significantly lower score in multivariable analysis; meanwhile, there was no difference in score for focal segmental glomerulosclerosis, membranoproliferative glomerulonephritis, and lupus nephritis. Notably, only pauci-immune glomerulonephritis was associated with a higher score than diabetic glomerular nephropathy.

## 4. Discussion

The present study describes the epidemiology of glomerular diseases diagnosed by kidney biopsy in the largest tertiary nephrology center from Romania in a ten-year time frame (2008–2017). More than half of the included patients were diagnosed with primary glomerular disease. IgA nephropathy was the most frequent diagnosis, followed by membranous nephropathy.

Chronic histopathological changes, typically irreversible, are as critical as the primary diagnosis in managing glomerular nephropathies. The proposed renal histopathological prognostic score, which assesses common chronic lesions, correlated with lower eGFR and higher proteinuria. Notably, patients with diabetic glomerular nephropathy recorded the highest scores.

The frequency of glomerulopathies in our center aligns with reports from other European countries, yet our center shows a lower rate of kidney biopsies relative to the addressed population. This occurs despite an increasing trend in biopsies over the decade analyzed and the broadening of biopsy criteria to include secondary nephropathies, particularly among diabetic and elderly patients. Nephrotic syndrome was the primary reason for biopsy in our study population, contrasting with other European studies where chronic nephritic syndrome or asymptomatic urinary abnormalities were more common [3]. This difference suggests that glomerular diseases may be recognized later in our context, often only when symptoms become clinically apparent.

In our center, the mean age of adults undergoing a kidney biopsy was 50 years, aligning with reports from other European studies (Table 5). European registries show a trend towards older ages at the time of the kidney biopsy, yet in our study, only 18% of the patients were older than 65 years.

The prevalence of nephrotic syndrome in glomerular diseases increased with age, whereas nephritic syndrome showed a decline. This pattern aligns with the age-related epidemiology observed for specific conditions. IgA nephropathy is associated with nephritic syndrome, while amyloidosis, minimal change disease and membranous nephropathy are linked with nephrotic syndrome, reflecting age-dependent disease distributions.

Male patients constituted the majority at 57%, consistent with prior European findings ranging from 50% to 65%. Similarly, the increased prevalence of female patients in cases of lupus nephritis, Alport syndrome/thin basement membrane disease, and amyloidosis aligned with observations from the Flemish Collaborative Glomerulonephritis Group registry [21].

We focused our study on the indications for kidney biopsy within our regional context, highlighting variations that may arise due to factors like age and local clinical resources, rather than the overall incidence of glomerular diseases. This approach provides insight into local diagnostic strategies, emphasizing their alignment with available therapeutic resources, rather than presenting a comprehensive epidemiological analysis of kidney diseases. Moreover, comparing disease frequencies and incidences across kidney biopsy registries is complex due to several issues. Not all registries report both metrics; frequencies are often based on specific subgroups rather than all biopsies, and there is significant variability in the diagnostic terms used. Additionally, past reviews have employed outdated terms without always adjusting frequencies against the total biopsy count.

To address these issues, for comparative reasons, we analyzed the incidence and frequency distribution of glomerular diseases at our center, drawing on the methodology of the Flemish Collaborative Glomerulonephritis Group registry [21]. The study published by the registry included reports from Europe, with reference populations exceeding 1 million. Following their approach, we recalculated some glomerular disease frequencies based on the total number of adult biopsies (Table 5).

IgA nephropathy emerged as the most common glomerular disease identified through kidney biopsy, aligning with numerous reports from Europe (Table 5), Asia, and the USA. Notably, IgA nephropathy patients in our study were often diagnosed at advanced stages of CKD, with a median eGFR of 38.4 mL/min—a contrast to the findings of many similar epidemiological studies in Europe. This significant observation affects both renal and overall patient outcomes, as it suggests that close to half of the patients are beyond the “point of no return” in the progression of this disease.

The literature suggests that geographic variation in the prevalence of IgAN across European countries might be influenced by genetic factors, dietary habits, and environmental exposures [26,27,28,29]. For instance, the higher frequency of approximately 20% in Italy, Romania, Poland, the Czech Republic, and Lithuania could be linked to shared genetic backgrounds or common dietary practices that enhance immune responses leading to IgAN [26]. This aligns with studies on geographic variation in genetic risk for IgAN [29]. Conversely, the lower rates of 7–9% in Serbia and 14% in Spain might reflect genetic protective factors or differences in environmental exposure. Variations in diagnostic practices and healthcare access could also impact these prevalence rates. Further research is required to elucidate the complex interplay of factors contributing to the geographic variations in IgAN occurrence.

In our center, we observed one of the highest incidences of diabetic glomerular nephropathy in Europe at 10% (Table 5). This reflects a growing interest in performing kidney biopsies on patients with diabetes mellitus. This approach is crucial because up to one-third of diabetic patients with suspected diabetic kidney disease may have another glomerular disease coexisting with diabetic glomerular nephropathy or a different glomerulopathy [30]. Moreover, the severity of lesions evaluated with the Renal Pathology Society classification for diabetic glomerular nephropathy, interstitial fibrosis and tubular atrophy score and glomerular basement membrane thickness can have utility in predicting kidney survival [31].

The most frequent causes of primary nephrotic syndrome in our cohort were membranous nephropathy and minimal change disease. Interestingly, we observed one of the highest frequencies of both membranous nephropathy and minimal change disease in Europe. For membranous nephropathy, the difference in incidence may reflect a decrease in kidney biopsy procedures in cases positive for antiphospholipase A2-receptor antibodies (Table 5).

Clinically, patients with these conditions presented with similar median age and severity of nephrotic syndrome (Table 3). However, patients with membranous nephropathy had slightly better eGFR at diagnosis, suggesting a lower prevalence of acute kidney injury compared to minimal change disease.

Chronicity lesions observed in native kidney biopsies predict renal outcomes and could influence treatment approaches across various glomerular diseases [17,18]. To provide a more consistent assessment of chronic changes in native kidney biopsies, irrespective of the diagnosis, the renal histopathological prognostic score was introduced as a standardized pathology scoring system for glomerulopathies [18]. This score’s prognostic accuracy was confirmed in a broad cohort encompassing diverse glomerular diseases in native kidney biopsies, demonstrating its ability to predict outcomes comparably to the more intricate Mayo Clinic Chronicity Score [17,18].

Older age appeared to be associated with a higher degree of chronicity, but after correction for potential confounders in multivariable analysis, chronicity was not related to older age.

Proteinuria may reflect both disease activity and/or chronicity, likely explaining the relationship observed in our model. As expected, lower eGFR was associated with an increased renal histopathological prognostic score.

Diabetic glomerular nephropathy exhibited the highest score and the strongest association with a higher chronicity grade compared to other glomerular diseases, reflecting the chronicity burden induced by diabetes mellitus across all kidney compartments. However, pauci-immune glomerulonephritis scored higher than diabetic glomerular nephropathy, potentially mirroring the epidemiology of ANCA vasculitis, which is more common in older patients with chronic lesions and features extracapillary proliferation. This discrepancy may also reflect the variations in biopsy practices in our center.

This study faces several limitations. Its retrospective, observational design may introduce selection bias and affect the representativeness of the study population due to the exclusion of certain patient groups and reliance on historical medical records, potentially leading to data inaccuracies. Additionally, using only data from the time of biopsy ignores any subsequent clinical changes.

The study’s dependency on a single center and the subjective nature of the renal histopathological prognostic score may also introduce inconsistencies in diagnosis and scoring. Furthermore, the retrospective classification of clinical syndromes might not capture the full clinical complexity of each case.

In conclusion, this decade-long study in Romania highlights IgA nephropathy as the most frequent glomerular disease diagnosed by kidney biopsy. Diabetic glomerular nephropathy and lupus nephritis were identified as the most common secondary forms. The renal histopathological prognostic score, notably high in diabetic glomerular nephropathy patients, was correlated with lower eGFR and higher proteinuria, underlining its clinical relevance. These insights are important for refining kidney disease management strategies within the region.

## Figures and Tables

**Table 1:** Definitions of clinical nephrological syndromes.

Nephrotic syndrome	
Proteinuria, 24 h collection (g/day)	≥3.5
Serum albumin (g/dL)	<3.5
Nephritic syndrome	
Dysmorphic hematuria	Yes
Proteinuria, 24 h collection (g/day)	≥1.0
Kidney injury	AKI/CKD
Nephrotic syndrome	No
Chronic kidney disease	
KDIGO criteria for CKD	Yes
Nephrotic syndrome	No
Nephritic syndrome	No
Acute kidney injury	
KDIGO criteria for AKI	Yes
Nephrotic syndrome	No
Nephritic syndrome	No
KDIGO criteria for CKD	No
Asymptomatic urinary abnormalities *	
Hematuria	Yes
Proteinuria, 24 h collection (g/day)	<1.0
CKD/AKI	No
Nephrotic syndrome	No
Nephritic syndrome	No

AKI, acute kidney injury; CKD, chronic kidney disease; KDIGO, Kidney Disease Improving Global Outcome (https://kdigo.org, accessed on 5 January 2020). * Oligosymptomatic urinary abnormalities.

**Table 2:** Patients’ data at the time when the kidney biopsy was performed.

	N = 1254
Age, years ^#^	
Male sex, %	57
Clinical presentation, %	
Nephrotic syndrome	46
Nephritic syndrome	37
AUA	4
AKI	1
CKD	12
Etiology, n (%)	
Primary GN	698 (54)
IgA nephropathy	256 (20)
Membranous nephropathy	198 (16)
Minimal change disease	130 (10)
pFSGS	44 (4)
MPGN	59 (5)
Fibrillary and imunotactoid GN	11 (1)
Secondary GN	556 (46)
Lupus nephritis	111 (9)
Diabetic glomerular nephropathy	128 (10)
sFSGS	47 (4)
Amyloidosis	87 (7)
LCDD	8 (1)
Pauci-immune GN	60 (5)
Thin basement membrane GN	40 (3)
Alport syndrome	24 (2)
Infection-related immune-complex GN	35 (3)
Thrombotic microangiopathy	6 (0)
Anti-GBM GN	9 (1)
Fabry disease	1 (0)
Arterial hypertension, %	59
Blood pressure, mmHg *	140 (120–160)/80 (80–90)
Serum creatinine, mg/dL *	1.56 (1.08–2.80)
eGFR, mL/min *	41.9 (21.4–65.0)
Proteinuria, g/day *	3.00 (1.12–5.70)
Serum albumin, g/dL *	3.65 (2.96–4.20)
Total serum protein, g/dL *	6.40 (5.50–7.20)
Hemoglobin, g/dL ^#^	12.3 ± 4.5
C-reactive protein, mg/L *	4.0 (1.0–11.0)
Serum cholesterol, mg/dL *	235.0 (183.0–301.0)
Serum triglycerides, mg/dL *	181.0 (125.0–263.0)
Hematuria, h/HPF *	45 (5–210)
Dysmorphic hematuria, %	48
Number of glomeruli per KB *	9 (5–11)
Pathology lesions, %	
Glomerulosclerosis	49
Tubular atrophy	25
Interstitial fibrosis	29
Crescents	20
Arteriolosclerosis	24
Renal histopathological prognostic score *	1 (0–2)

* Denotes median (IQR); ^#^ denotes mean ± SD; AUA, asymptomatic urinary abnormalities; AKI, acute kidney injury; CKD, chronic kidney disease; eGFR, estimated glomerular filtration rate; FSGS, focal segmental glomerulosclerosis; GBM, glomerular basement membrane; GN, glomerulonephritis; HPF, high power field; IgA, immunoglobulin A; KB, kidney biopsy; MPGN, membranoproliferative glomerulonephritis; LCDD, light chain deposition disease; p, primary; s, secondary.

**Table 3:** Patients’ data at the time when the kidney biopsy was performed for the first seven most frequent glomerular diseases (80% of the studied population).

	IgAN n = 256	MN n = 198	MCD n = 130	Lupus GN n = 111	Diabetic GN n = 128	Amyloidosis n = 87	TBM/Alport n = 64
Age, years *	44 (34–57)	56 (42–67)	50 (38–63)	37 (29–51)	56 (49–63)	63 (52–68)	40 (31–50)
Male sex, %	71	62	53	26	68	48	30
Clinical presentation, %							
Nephrotic syndrome	9	92	82	50	31	84	4
Nephritic syndrome	79	2	10	30	10	6	69
AUA	2	2	4	6	9	0	14
AKI	1	1	3	0	4	0	0
CKD	9	3	1	14	46	10	13
Arterial hypertension, %	70	49	40	53	81	35	50
eGFR, mL/min *	38.4 (22.4–57.1)	64.1 (48.0–79.3)	56.4 (33.6–82.7)	50.2 (24.4–74.0)	25.9 (17.3–42.3)	40.3 (19.0–58.4)	59.0 (47.3–79.2)
Proteinuria, g/day *	1.5 (0.6–3.3)	5.5 (3.2–8.3)	4.3 (2.0–7.5)	2.8 (1.0–4.6)	3.8 (3.3–4.2)	5.2 (3.6–7.7)	1.0 (0.0–2.1)
Serum albumin, g/dL *	4.2 (3.8–4.5)	3.0 (2.4–3.4)	2.9 (2.4–3.6)	3.4 (2.9–3.9)	3.8 (3.3–4.2)	2.9 (2.3–3.4)	4.4 (4.1–4.7)
Hematuria, h/HPF *	180 (40–235)	18 (5–65)	15 (5–68)	160 (15–230)	5 (5–55)	5 (5–35)	80 (19–220)
Hemoglobin, g/dL *	13.0 (11.0–14.5)	13.2 (11.9–14.6)	13.7 (12.0–15.1)	10.8 (9.4–12.2)	11.1 (9.8–13.0)	12.0 (10.2–13.6)	12.8 (11.9–14.3)
C-reactive protein, mg/L *	3.0 (1.0–8.0)	3.0 (1.0–7.0)	3.0 (1.0–6.0)	4.0 (2.0–10.0)	5.0 (2.0–13.0)	6.0 (2.0–25.0)	2.0 (1.0–5.0)
Renal histopathological prognostic score *	1 (1–2)	0 (0–1)	0 (0–1)	1 (1–2)	2 (1–3)	1 (0–2)	0 (0–1)

* Denotes median (IQR); AKI, acute kidney injury; AUA, asymptomatic urinary abnormalities; CKD, chronic kidney disease; IgAN, immunoglobulin A nephropathy; GN, glomerulonephritis; MN, membranous nephropathy, MCD, minimal change disease; TBM, thin basement membrane.

**Table 4:** Univariate and multivariable linear regression analysis of clinical characteristics and renal histopathological prognostic score.

Variables	Univariate			Multivariable		
	β	95%CI	*p*	β	95%CI	*p*
Sex						
Male (reference)						
Female	−0.118	−0.251, 0.016	0.08	−0.103	−0.216, 0.009	0.07
Age (years)	0.010	0.005, 0.014	<0.001	−0.001	−0.005, 0.003	0.7
eGFR (mL/min)	−0.020	−0.022, −0.018	<0.001	−0.014	−0.016, −0.012	<0.001
Proteinuria (g/day)	−0.001	−0.014, 0.012	0.8	0.014	0.002, 0.026	0.01
Diagnosis						
Diabetic glomerular nephropathy (reference)						
IgA nephropathy	−0.37	−0.59, −0.15	<0.001	−0.20	−0.42, 0.00	0.05
Membranous nephropathy	−1.28	−1.51, −1.05	<0.001	−0.83	−1.06, −0.60	<0.001
Minimal change disease	−1.43	−1.68, −1.18	<0.001	−1.05	−1.30, −0.81	<0.001
FSGS	−0.28	−0.56, −0.03	0.04	−0.06	−0.32, 0.19	0.6
MPGN	−0.24	−0.56, 0,07	0.1	−0.18	−0.48, 0.11	0.2
Lupus nephritis	−0.46	−0.72, −0.19	<0.001	−0.11	−0.37, 0.14	0.3
Amyloidosis	−0.87	−1.16, −0.59	<0.001	−0.74	−1.01, −0.47	<0.001
Pauci-immune GN	0.86	0.55, 1.16	<0.001	0.65	0.36, 0.94	<0.001
Thin basement membrane GN/Alport syndrome	−1.16	−1.47, −0.85	<0.001	−0.64	−0.95, −0.34	<0.001
Infection-related immune-complex GN	−0.42	−0.80, −0.03	0.03	−0.49	−0.85, −0.12	0.009
Other	−0.09	−0.52, 0.34	0.6	−0.11	−0.51, 0.29	0.5

FSGS, focal segmental glomerulosclerosis; GN, MPGN, membranoproliferative glomerulonephritis; GN, glomerulonephritis.

**Table 5:** Comparison of glomerular disease frequency and incidence diagnosed by kidney biopsy in Europe—adaptated from Laurens et al. [21]. The included studies face several limitations: not all registries report both frequency and incidence; disease frequencies are often calculated within specific subgroups rather than across all biopsies, and there is a wide variation in the diagnostic terminology used. Moreover, earlier reviews relied on outdated diagnostic terms and did not always calculate frequencies relative to the total number of biopsies. The table covers the incidence and frequency of glomerular diseases in 13 European studies, with some frequencies recalculated for either all adult biopsies or a combination of adult and pediatric biopsies, as permitted by data [21]. The scope of these studies ranges from international to single-center, each catering to populations exceeding 1 million.

Study	Region/ Population	Timeframe	Kidney Biopsies (N)	Age (years)/ Male (%)	A/P	IgAN (%)	MN (%)	MCD (%)	FSGS (%)	MPGN (%)	LN (%)	AAV (%)	Amy (%)	DKD (%)
Schena et al., 1997 [11]	Italy/56.9 million	1987–1993	13,835	NA/65	A + P	21.1	12.4	4.7	7.1	4	6.7	3.4	2.7	NA
Rivera et al., 2002 [10]	Spain/40 million	1994–1999	6525	NA/60	A	14.6	9.9	6.8	8	3.9	8.8	7.8	4.3	1
Covic et al., 2006 [16]	Romania/6 million	1995–2004	606	38.5/52	A	19.1	7.4	5.6	7.6	19.5	7.8	5.4	3.1	NA
Wirta et al., 2008 [22]	Finland/0.8 million	1980–2000	3310	38–52/59	A + P	21.7	2.4	3.1	2.4	2.4	NA	NA	NA	NA
Naumovic et al., 2009 [14]	Serbia/7.5 million	1987–2006	1626	39.1/51	A	7.7	12.6	4.8	12.1	6.7	10.1	2.3	1.4	NA
Brazdziute et al., 2015 [4]	Lithuania/3.4 million	1994–2012	3213	43.2/58.4	A + P	20.2	4.7	4.9	7.8	7.4	2.3	NA	6.6	1.2
Maxinerova et al., 2015 [8]	Czech Rep/10.3 million	1994–2011	9051	44.5/58	A	20.5	7.1	6.1	6.9	3.2	7.1	5.7	3.1	4.1
Perkowska-Ptasinka et al., 2017 [23]	Poland/38.5 million	2009–2014	7349	NA/54	A	20	11.2	5.5	15	4.6	8.4	5.5	4.5	3.7
Brkovic et al., 2018 [24]	Serbia/7.2 million	2007–2014	665	42/50	A	9	17.3	3.8	12.5	4.8	17.7	4.4	0.9	NA
O’Shaughnessy et al., 2018 [3]	Europe International/NA	2006–2018	19,302	48/56	A + P	16.2	9.8	5	11.6	2.8	7.8	6.2	3.4	5.4
Lopez-Gomez et al., 2020 [25]	Spain/40 million	1994–2019	25,440	50/60	A	14.6	9.9	6.8	8	3.9	8.7	6.8	3.8	4.8
Laurens et al., 2022 [21]	Belgium/5.3 million	2017–2019	2054	61.1/62	A	17.3	5.5	4.7	9.3	0.7	4.2	7.2	3.4	7.5
Current study	Romania/8.5 million	2008–2017	1254	50/57	A	20	16	10	8	5	9	5	7	10

A, adult; AAV, ANCA associated vasculitis; Amy, amyloidosis; DKD, diabetic kidney disease; FSGS, focal segmental glomerulosclerosis; IgAN, immunoglobulin A nephropathy; LN, lupus nephritis; MCD, minimal change disease; MPGN, membranoproliferative glomerulonephritis; MN, membranous nephropathy; NA, not assessed; P, pediatric; TBM, thin basement membrane.

## Data Availability

The data underlying this article will be shared on reasonable request by the corresponding author.

## Supplementary Materials

The following supporting information can be downloaded at: https://www.mdpi.com/article/10.3390/biomedicines12061143/s1, Table S1: Patients data at the time when kidney biopsy was performed; comparison between clinical presentations.

## Abbreviations

A, adult; AAV, ANCA associated vasculitis; Alp, Alport syndrome; Amy, amyloidosis; AUA, asymptomatic urinary abnormalities; AKI, acute kidney injury; CKD, chronic kidney disease; DN, diabetic glomerular nephropathy; eGFR, estimated glomerular filtration rate; FC, fibrocellular/fibrous crescents; FID, fibrillary and imunotactoid GN; FSGS, focal segmental glomerulosclerosis; GD, glomerular diseases; GBM, glomerular basement membrane; GN, glomerulonephritis; GS, glomerulosclerosis; HPF, high power field; IF, interstitial fibrosis; IgA, immunoglobulin A; InfGN, infection-related immune-complex GN; IQR, interquartile range; KB, kidney biopsy; LCDD, light chain deposition disease; LN, lupus nephritis; MCD, minimal change disease; MPGN, membranoproliferative glomerulonephritis; MN, membranous nephropathy; P, pediatric; SD, standard deviation; TA, tubular atrophy; TBM, thin basement membrane.
