# Supplementary material for: Clinicopathological Characteristics and Disease Chronicity in Glomerular Diseases: A Decade-Long Study at Romania’s Largest Kidney Biopsy Reference Center
Source: Biomedicines. 2024 May 22;12(6):1143. doi: 10.3390/biomedicines12061143 (PMC11200787; doi:10.3390/biomedicines12061143)
Supplement: Supplementary file 1 [file biomedicines-12-01143-s001.zip › biomedicines-2992484-supplementary.pdf]

Supplementary Table S1: Patients data at the time when kidney biopsy was performed; comparison between clinical presentations

	Nephrotic synd n=570	Nephritic synd n=463	AUA n=46	AKI n=11	CKD n=164
Age, years*	53 (38-64)	47 (36-59)	44 (35-57)	51 (50-67)	53 (43-62)
Male sex, %*	45	42	46	82	40
Etiology, n (%)*					
IgA nephropathy	24 (4)	202 (44)	4 (9)	0 (0)	25 (15)
Membranous nephropathy	183 (32)	6 (1)	5 (11)	0 (0)	4 (2)
Minimal change disease	106 (19)	15 (3)	5 (11)	4 (28)	1 (1)
Lupus nephritis	56 (10)	33 (7)	7 (14)	0 (0)	15 (9)
Diabetic glomerular nephropathy	39 (7)	13 (3)	12 (26)	5 (46)	59 (36)
TBM/Alport	3 (1)	44 (10)	9 (20)	0 (0)	8 (5)
Amyloidosis	73 (13)	5 (1)	0 (0)	0 (0)	9 (6)
Pauci-immune GN	5 (1)	59 (13)	0 (0)	3 (26)	4 (2)
Other	81 (13)	86 (18)	4 (9)	0 (0)	39 (24)
Arterial hypertension, %*	50	67	48	27	74
eGFR, mL/min*	52.5 (28.7-75.0)	35.8 (17.0-55.2)	63.4 (52.3-81.2)	12.6 (6.8-21.8)	27.2 (15.8-41.7)
Proteinuria, g/day*	5.20 (3.8-8.00)	1.45 (0.60-3.06)	1.83 (0.59-3.50)	0.02 (0.00-0.52)	1.70 (0.45-3.94)
Serum albumin, g/dL*	2.9 (2.4-3.4)	4.1 (3.7-4.4)	4.2 (3.9-4.7)	4.2 (3.3-4.7)	4.0 (3.6-4.4)
Hemoglobin, g/dL	12.6 (10.7-14.3)	12.0 (9.9-13.7)	13.2 (11.7-15.1)	11.5 (10.2-12.3)	11.3 (9.5-13.2)
C-reactive protein, mg/L*	3.0 (1.0-8.0)	4.0 (1.5-12.5)	2.0 (1.0-5.0)	31.0 (8.0-64.0)	5.0 (1.5-15.0)
Serum cholesterol, mg/dL*	291.0 (226.0-376.0)	208.0 (171.0-246.0)	227.5 (183.0-258.0)	174.0 (136.0-225.0)	197.0 (160.0-246.0)
Serum triglycerides, mg/dL*	291.0 (226.0-376.0)	156.0 (107.0-218.0)	144.0 (111.0-254.0)	200.0 (125.0-291.0)	181.0 (120.0-262.0)
Hematuria, h/HPF*	30 (5-170)	190 (40-250)	5 (5-60)	5 (5-230)	10 (5-33)
Renal histopathological prognostic score*	1 (0-1)	1 (1-2)	1 (0-1)	0 (0-1)	2 (1-3)

* significant statistical difference

AUA, asymptomatic urinary abnormalities; AKI, acute kidney injury; CKD, chronic kidney disease; eGFR, estimated glomerular filtration rate; GN, glomerulonephritis; HPF, high power field; IgA, immunoglobulin A; TBM, thin basement membrane
